# Abnormal Coagulation Function of Patients With COVID-19 Is Significantly Related to Hypocalcemia and Severe Inflammation

**DOI:** 10.3389/fmed.2021.638194

**Published:** 2021-06-16

**Authors:** Xu Qi, Hui Kong, Wenqiu Ding, Chaojie Wu, Ningfei Ji, Mao Huang, Tiantian Li, Xinyu Wang, Jingli Wen, Wenjuan Wu, Mingjie Wu, Chaolin Huang, Yu Li, Yun Liu, Jinhai Tang

**Affiliations:** ^1^Department of Respiratory Medicine, The First Affiliated Hospital of Nanjing Medical University, Nanjing, China; ^2^Division of Intensive Care Unit, Wuhan Jin Yin-tan Hospital, Wuhan, China; ^3^The Third Clinical Medical College, Nanjing University of Chinese Medicine, Nanjing, China; ^4^School of Medicine and Holistic Integrative Medicine, Nanjing University of Chinese Medicine, Nanjing, China

**Keywords:** COVID-19, metabolic disorder, coagulation function, hypocalcemia, inflammation

## Abstract

This study aimed to detect, analyze, and correlate the clinical characteristics, blood coagulation functions, blood calcium levels, and inflammatory factors in patients with mild and severe COVID-19 infections. The enrolled COVID-19 infected patients were from Wuhan Jin Yin-tan Hospital (17 cases, Wuhan, China), Suzhou Infectious Disease Hospital (87 cases, Suzhou, China), and Xuzhou Infectious Disease Hospital (14 cases, Xuzhou, China). After admission, basic information was collected; X-ray and chest CT images were obtained; and data from routine blood tests, liver and kidney function, myocardial enzymes, electrolytes, blood coagulation function, (erythrocyte sedimentation rate) ESR, C-reactive protein (CRP), IL-6, procalcitonin (PCT), calcitonin, and other laboratory tests were obtained. The patients were grouped according to the clinical classification method based on the pneumonia diagnosis and treatment plan for new coronavirus infection (trial version 7) in China. The measurements from mild (56 cases) and severe cases (51 cases) were compared and analyzed. Most COVID-19 patients presented with fever. Chest X-ray and CT images showed multiple patchy and ground glass opacities in the lungs of COVID 19 infected patients, especially in patients with severe cases. Compared with patients with mild infection, patients with severe infection were older (*p* = 0.023) and had a significant increase in AST and BUN. The levels of CK, LDH, CK-MB, proBNP, and Myo in patients with severe COVID-19 infection were also increased significantly compared to those in patients with mild cases. Patients with severe COVID-19 infections presented coagulation dysfunction and increased D-dimer and fibrin degradation product (FDP) levels. Severe COVID-19 patients had low serum calcium ion (Ca^2+^) concentrations and high calcitonin and PCT levels and exhibited serious systemic inflammation. Ca^2+^ in COVID-19 patients was significantly negatively correlated with PCT, calcitonin, D-dimer, PFDP, ESR, CRP and IL-6. D-dimer in COVID-19 patients was a significantly positively correlated with CRP and IL-6. In conclusion, patients with severe COVID-19 infection presented significant metabolic dysfunction and abnormal blood coagulation, a sharp increase in inflammatory factors and calcitonin and procalcitonin levels, and a significant decrease in Ca^2+^. Decreased Ca^2+^ and coagulation dysfunction in COVID-19 patients were significantly correlated with each other and with inflammatory factors.

## Background

It has been nearly a year since the discovery of the novel coronavirus—COVID-19, which has become a global-scale disaster event. COVID-19 infected patients can develop serious pneumonia and metabolic disorders (1), acute respiratory distress syndrome (ARDS), multiple organ dysfunction (MODS), and even septic shock and death (2–4).

Dysfunction of the coagulation/fibrinolysis system is an important pathophysiological feature of COVID-19 patients, and it is related to the inflammatory cascade induced by viral infection (5–7). The diffuse intravascular coagulation (DIC) induced by severe infections often becomes a decisive factor in the death of patients with severe infection (8–11).

To date, many studies have revealed dysfunction of the coagulation/fibrinolysis system in COVID-19 infected patients. Although a review titled “Coagulation and anticoagulation in COVID-19” was recently published (12), the specific relationship between the coagulation function and inflammation in patients with mild and severe COVID-19 infection, as well as the correlation between coagulation function and serum Ca^2+^ [which was abnormally decreased in patients with severe COVID-19 infection (13)], has not yet been determined.

Therefore, we analyzed basic information, X-ray and chest CT images, routine blood tests, liver and kidney function, myocardial enzymes, electrolytes, blood coagulation function, erythrocyte sedimentation rate (ESR), C-reactive protein (CRP), IL-6, procalcitonin (PCT), and calcitonin in mild and severe cases of COVID-19. We focused on the coagulation function of these cases and compared and analyzed the correlations among decreased Ca^2+^, coagulation disorder and other test results, including systemic inflammation and metabolic disorders, in mild and severe COVID-19 infected patients.

In this study, the above indicators and correlations of COVID-19 patients with different severity were analyzed to further explore the role of coagulation function in COVID-19 infection, and in especial, to reveal the role of hypocalcemia in severe COVID-19 infection. Our present study suggests that coagulation function and serum Ca^2+^ concentration should be closely supervised when treating patients with COVID-19 infection.

## Materials and Methods

### COVID-19 Infection Diagnosis

All cases met the diagnostic criteria of “The Pneumonia diagnosis and treatment plan for new coronavirus infection (trial version 7)” of China (14) (http://www.nhc.gov.cn/yzygj/s7653p/202003/46c9294a7dfe4cef80dc7f5912eb1989.shtml). All cases had the corresponding clinical manifestations: fever, imaging features of pneumonia, normal or decreased white blood cell count or decreased lymphocyte count in the early stage of COVID-19 infection, and pathogenic evidence of a positive SARS-CoV-2 gene.

For SARS-CoV-2 gene detection, throat swab samples were collected from patients and immediately tested by using a transcription polymerase chain reaction (PCR) system to detect the SARS-CoV-2 gene. A reverse transcription polymerase chain reaction (RT-PCR) kit (Da'an Gene, Shenzhen, China) was used to detect SARS-CoV-2 conserved genes through the ABI7500 system (Roche). All samples that were positive for SARS-CoV-2 or highly homologous with known SARS-CoV-2 (15) were confirmed to be positive for COVID-19.

### COVID-19 Patients

The data for 107 cases were collected from three hospitals that received and cured COVID-19 patients from February 2 to June 25, 2020: Wuhan Jin Yin-tan Hospital (17 cases), Suzhou Infectious Disease Hospital (76 cases), and Xuzhou Infectious Diseases Hospital (14 cases). These data include basic clinical records, laboratory results, and X-ray and computed tomography (CT) scan images of the chest. The basic clinical records included the general information of the patients, such as the age, sex, contact history, admission temperature, disease course, and comorbidities (hypertension or diabetes). The laboratory test results included the blood cell count, liver and kidney functions, electrolytes, myocardial enzymes, coagulation function, ESR, CRP, IL-6, PCT, and calcitonin levels. Death cases were excluded, and all patients were eventually discharged from the hospital. Patients were grouped according to the clinical classification method of “The Pneumonia diagnosis and treatment plan for new coronavirus infection (trial version 7).” A total of 107 patients were divided into two groups according to the severity of the disease: the mild group (56 cases) and the severe group (51 cases). The study design was approved by the hospital ethics committee of the three hospitals.

### Laboratory Data Analysis

Blood samples from patients were used for laboratory tests. A DxH 800 Coulter blood cell analyzer (Beckmann, America) was used to detect the patient's blood cell classification and ESR. Serum biochemical tests, which reflect the patient's liver and kidney function, electrolytes, and myocardial enzymes, including ALT, AST, ALP, ALB, CRP, TBIL, DBIL, BUN, Cr, TnT, CK, LDH, CK-MB, proBNP, Myo, Na^+^, K^+^, Cl^−^, and Ca^2+^ were carried out by an automatic biochemistry analyzer system (Roche, Germany). IL-6, PCT and calcitonin levels were analyzed by a CL-2000i chemiluminescence immunoassay system (Mindray, Shenzhen, China). An ACL TOP 700 hemostasis test system (Wolfen, USA) was used to detect coagulation indicators.

### Statistical Analysis

The SPSS 23.0 statistical software was used to analyze the data. Firstly, the Levene variance equality test was used to determine whether the variance within the groups was equality according to the *F*-value. After that, an independent sample *T*-test was performed, and the *p*-value under the assumption of equal variance or unequal variance were all analyzed. The reasonable statistical results of *p*-value were selected according to the *F*-value of Levene variance equality test. The measurement data are expressed as the mean ± standard deviation. A value of *p* < 0.05 was considered statistically significant. Pearson correlation analysis between different indexes was carried out using SPSS 23.0 statistical software. Some indexes were regarded as a same variable data set and Canonical correlation was used to analyze the correlation between these data sets using SPSS 23.0 statistical software. GraphPad Prism 7.0 (San Diego, CA, USA) was used to perform the correlation analysis of the data. The correlation coefficient and linear regression were jointly used to analyze the correlation of the indicated data.

## Results

### Changes in Clinical Characteristics and Blood Cell Counts of Patients With Mild and Severe COVID-19 Infection

**Figure 1:**
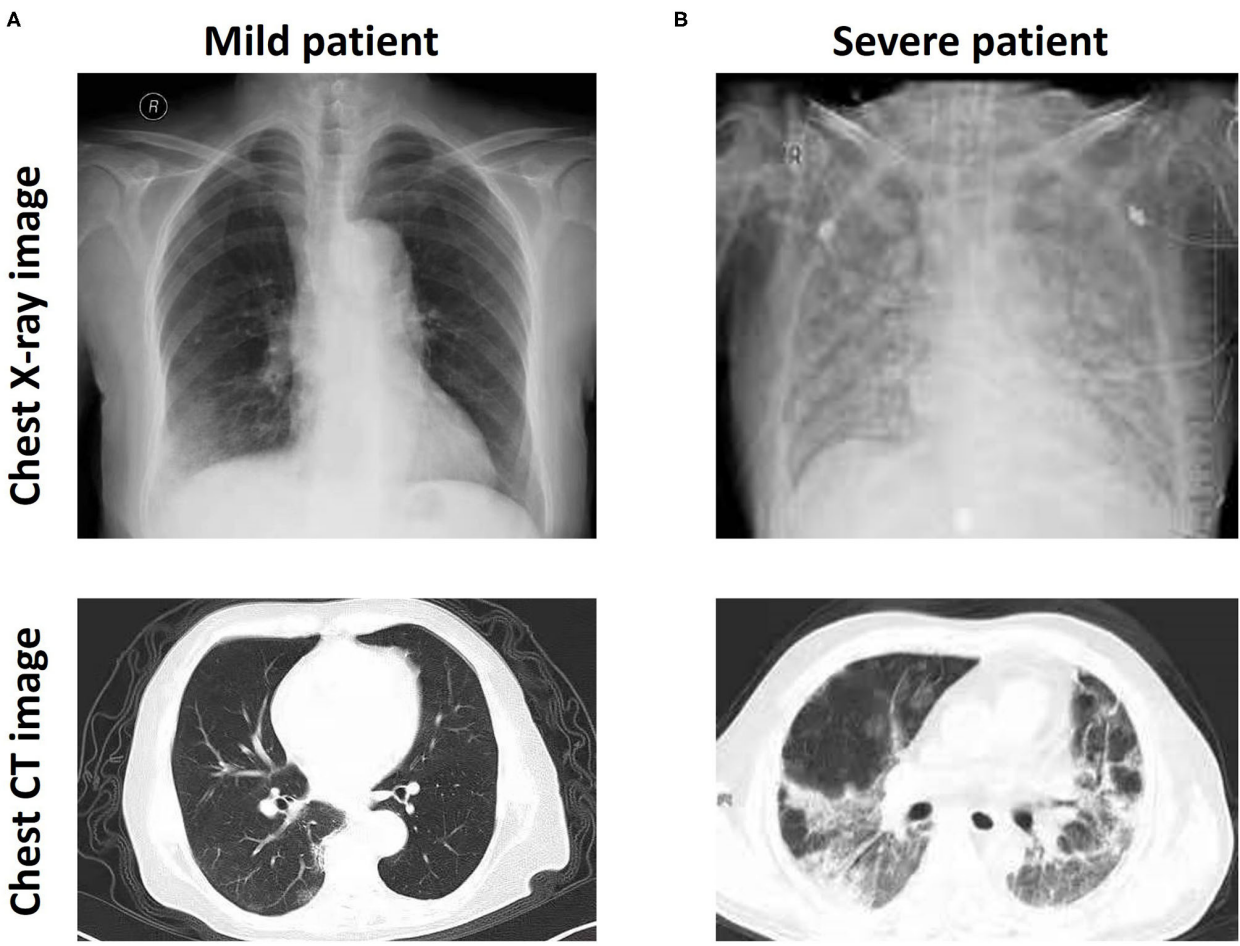
CT and bedside chest X-ray images of patients with mild and severe COVID-19. Bedside chest X-ray and chest CT images showing multiple patchy shadows and ground glass opacity in the lungs of patients with mild **(A)** and severe **(B)** COVID-19.

All patients were diagnosed with positive SARS-CoV-2 gene expression by RT-PCR. Most patients presented with fever (the average body temperature of all patients was 37.79 ± 1.08°C). Chest X-ray and CT images showed multiple patchy and ground glass opacities in the lungs (Figure 1A), thus revealing a wide range of lesions and pulmonary damage, especially in patients with severe COVID-19 infection (Figure 1B). The average age of 56 patients with mild disease was 46.16, with 31 males and 25 females in the group, and the average age of 51 patients with severe infection was 51.82, with 23 males and 28 females in the group. More than 98% of the patients (105/107) had a contact history. In total, 12 patients had other diseases, such as hypertension and diabetes. Patients with severe disease were older on average than those with mild disease, but there were no significant differences observed in the other characteristics, including height, weight, admission temperature, and the duration of infection (Table 1).

**Table 1:** Baseline characteristics in patients with mild and severe COVID-19 infection.

	**All patients (*n* = 107)**	**Patients with mild disease (*n* = 56)**	**Patients with severe disease (*n* = 51)**	***p*-value**	***F***
Age	48.86 ± 10.75	46.16 ± 10.48	51.82 ± 10.14	0.006	0.294
**Gender**					
Male	54 (50.46%)	31 (55.35%)	23 (45.09%)		
Female	53 (49.53%)	25 (44.62%)	28 (54.90)		
Height (cm)	166.73 ± 8.08	165.73 ± 7.48	167.82 ± 8.47	0.182	1.409
Weight (kg)	66.47 ± 11.43	66.95 ± 11.65	65.94 ± 11.05	0.652	0.964
Admission Temperature (°C)	37.79 ± 1.08	37.88 ± 1.12	37.69 ± 1.01	0.363	1.481
**Contact history**					
Yes	105 (98.13%)	54 (96.42)	51 (100%)		
No	2 (1.86%)	2 (3.57%)	0 (0%)		
Disease course (day)	11.11 ± 6.05	11.2 ± 6.09	11.02 ± 5.95	0.881	0.265
**Complication**					
Diabetes	6 (5.60%)	4 (7.14%)	2 (3.92%)		
Hypertension	7 (6.54%)	2 (3.57%)	5 (9.80%)		

Compared with the normal range, the number of white blood cells in patients with severe COVID-19 infection increased significantly (7.69 ± 1.61 × 10^9^/L, Normal range 4.3–5.8 × 10^9^/L), while the white blood cells of patients with mild infection (4.98 ± 0.55 × 10^9^/L) were still within the normal range. The increase in the level of white blood cells in the severe group was attributed to the increase in the level of neutrophils. The level of neutrophils was significantly increased in the blood of patients with severe cases (3.59 ± 2.53 × 10^9^/L vs. 5.72 ± 2.76 × 10^9^/L) compared with mild cases. The level of lymphocytes in patients with severe infection was lower than the normal range (1.03 ± 0.74 × 10^9^/L, normal range 1.1–3.2 × 10^9^/L) and was also significantly lower than the levels in patients with mild infection (1.32 ± 0.57 × 10^9^/L vs. 1.03 ± 0.74 × 10^9^/L; Table 2).

**Table 2:** Comparison of blood cell counts between patients with mild and severe COVID-19 infection.

	**Normal range**	**Patients with mild disease (*n* = 30)**	**Patients with severe disease (*n* = 30)**	***p*-value**	***F***
White blood cells (10^9^/L)	4.3–5.8	4.98 ± 0.55	7.69 ± 1.61	<0.001	58.146
Red blood cells (10^12^/L)	3.5–9.5	5.42 ± 2.49	5.95 ± 3.13	0.337	2.529
Hemoglobin (g/L)	130–175	152.34 ± 19.91	154.45 ± 20.64	0.595	0.015
Platelets (10^9^/L)	125–350	184.36 ± 62.22	193.67 ± 64.4	0.453	1.279
Neutrophils (10^9^/L)	1.8–6.3	3.59 ± 2.53	5.72 ± 2.76	<0.001	0.771
Lymphocytes (10^9^/L)	1.1–3.2	1.32 ± 0.57	1.03 ± 0.74	0.026	1.707
Monocytes (10^9^/L)	0.1–0.6	0.42 ± 0.16	0.38 ± 0.14	0.251	1.479
Eosnophils (10^9^/L)	0.02–0.52	0.02 ± 0.03	0.02 ± 0.06	0.914	0.120

### Patients With Mild and Severe COVID-19 Infection Present Varying Degrees of Metabolic Disorders and Abnormal Biochemical Tests

We observed a sharp increase in neutrophil levels in patients with severe COVID-19 infection, which may suggest that a very pronounced inflammatory response has occurred and disrupted the metabolism and function of multiple organs in the body (16). Therefore, we next evaluated the liver and kidney functions, myocardial enzymes, and electrolytes of the two groups of patients by using laboratory biochemical tests.

The liver and kidney function indexes of patients with severe disease were compared with those of patients with mild disease. For liver function, patients with severe disease had significantly increased levels of ALT, AST, ALP, TBIL, and DBIL (*p* < 0.001) and a higher level of BUN (*p* < 0.001). However, a considerable proportion of these liver and kidney function indicators were still within the normal range, including the levels of ALT, ALP, TBIL, DBIL, and BUN in patients with severe cases, which were significantly higher than those in patients with mild cases. It is worth noting that AST (46.06 ± 11.59 U/L, normal range 0.0–37.0 U/L) was extremely elevated in patients with severe disease, while ALB (28.61 ± 5.83 g/L, normal range 40–55 g/L) was extremely decreased, and both values were far from the normal range (Table 3).

**Table 3:** Comparison of blood biochemical tests and serum electrolytes between mild patients and patients with severe COVID-19 infection.

	**Normal range**	**Patients with mild disease (*n* = 30)**	**Patients with severe disease (*n* = 30)**	***p*-value**	***F***
**Liver related**					
ALT (U/L)	0.0–40.0	22.2 ± 5.8	28.37 ± 9.18	<0.001	20.995
AST (U/L)	0.0–37.0	20.45 ± 3.33	46.06 ± 11.59	<0.001	69.83
ALP (U/L)	45–125	62.48 ± 11.19	77.29 ± 14.93	<0.001	9.462
ALB (g/L)	40–55	44.64 ± 3.51	28.61 ± 5.83	<0.001	17.214
TBIL (μmol/L)	5.13–22.24	10.49 ± 2.47	13.01 ± 3.28	<0.001	6.064
DBIL (μmol/L)	1.70–8.55	3.67 ± 0.81	5.33 ± 1.39	<0.001	17.239
**Kidney related**					
BUN (mmol/L)	1.7–8.3	5.23 ± 1.48	6.32 ± 1.84	0.001	2.81
Cr (μmol/L)	36–132	58.75 ± 8.27	61.31 ± 7.95	0.109	0.073
**Heart related**					
TnT (ng/mL)	0–0.15	11.52 ± 5.68	13.69 ± 6.02	0.06	0.106
CK (U/L)	30–170	61.09 ± 19.52	94.69 ± 30.91	<0.001	16.535
LDH (U/L)	90–245	163.93 ± 34.48	520.78 ± 121.38	<0.001	53.866
CK-MB (ng/mL)	0–5	0.72 ± 0.12	1.88 ± 0.51	<0.001	68.471
proBNP (pg/mL)	0–125	52.95 ± 22.66	399.22 ± 184	<0.001	119.995
Myo (ng/mL)	0–70	26.77 ± 3.34	48.55 ± 7.7	<0.001	25.186
**Serum electrolytes**					
Na^+^ (mmol/L)	135–155	147.16 ± 10.23	145.46 ± 8.47	0.359	4.488
K^+^ (mmol/L)	3.5–5.5	4.3 ± 0.63	4.09 ± 0.61	0.079	0.033
Cl^−^ (mmol/L)	95–115	108.07 ± 4.94	107.8 ± 5.14	0.786	0.46
Ca^2+^ (mmol/L)	2.25–2.7	2.22 ± 0.07	1.91 ± 0.06	<0.001	0.149

More attention should be given to the heart function of patients with severe COVID-19 infection. Regarding myocardial enzymes, although the difference in TnT levels between mild and severe cases was not significant (11.52 ± 5.68 ng/ml vs. 13.69 ± 6.02 ng/ml, *p* = 0.06), the TnT levels of all patients were sharply increased and far exceeded the normal range (0–0.15 ng/ml). The levels of CK, LDH, CK-MB, proBNP, and Myo in severe cases were significantly increased compared with the levels of mild cases (*p* < 0.001). Among these indicators, the levels of LDH (520.78 ± 121.38 U/L, normal range 90–245 U/L) and proBNP (399.22 ± 184 pg/ml, normal range 0–125 pg/ml) increased tremendously in patients with severe disease and far exceeded the normal range (Table 3). This result suggested that patients with severe COVID-19 infection have abnormal heart function and may even have heart failure.

For serum electrolytes, the Na^+^, K^+^, and Cl^−^ levels in the mild and severe groups were within the normal range, but the level of Ca^2+^ in the severe group was significantly lower than that in the mild group (2.22 ± 0.07 mmol/L vs. 1.91 ± 0.06 mmol/L, *p* < 0.001), and it was also below the normal range (2.5–2.7 mmol/L) (Table 3). The decrease in Ca^2+^ may be related to the changes in myocardial enzymes in patients with severe COVID-19 infection.

### Patients With Severe COVID-19 Infection Present More Severe Coagulation Dysfunction

Ca^2+^ is an important factor in coagulation function (17), and it was significantly decreased in patients with severe COVID-19 infection. Thus, we examined indicators related to coagulation function in COVID-19 patients. Compared with patients with mild disease, patients with severe disease showed increased PT, including PT (s) (12.05 ± 0.63 s vs. 13.3 ± 1.36 s) and PT (%) (74.23 ± 9.19% vs. 95.59 ± 12.08%), but they also showed decreased fibrinogen (3.35 ± 0.75 g/L vs. 2.60 ± 0.59 g/L) and AT-3 (88.14 ± 4.29% vs. 73.73 ± 5.77%) activity reduction (*p* < 0.001). It is worth noting that the D-dimer and fibrin degradation products (FDPs) were within the normal range for patients with mild disease, while the D-dimer (5.54 ± 2.36 mg/L, normal range 0–1.5 mg/L) and FDP (70.15 ± 30.47 μg/ml, normal range 0–5 μg/ml) in patients with severe disease presented enormous abnormal increases that were several times or even dozens of times higher than the normal range (Table 4).

**Table 4:** Comparison of coagulation indicators between patients with mild and severe patients with COVID-19 infection.

	**Normal range**	**Patients with mild disease (*n* = 30)**	**Patients with severe disease (*n* = 30)**	***p*-value**	***F***
PT (s)	10.5–13.5	12.05 ± 0.63	13.3 ± 1.36	<0.001	59.902
PT (%)	75–125	74.23 ± 9.19	95.59 ± 12.08	<0.001	4.433
APTT (s)	21–37	26.96 ± 3.57	27.82 ± 3.72	0.228	0.141
TT (s)	13–21	16.62 ± 3.7	16.6 ± 3.24	0.967	1.793
INR	0.8–1.2	1 ± 0.12	1.02 ± 0.14	0.603	4.048
Fibrinogen (g/L)	2–4	3.35 ± 0.75	2.60 ± 0.59	<0.001	5.788
AT-3 (%)	75–125	88.14 ± 4.29	73.73 ± 5.77	<0.001	6.226
D-dimer (mg/L)	0–1.5	2.43 ± 0.65	5.54 ± 2.36	<0.001	99.096
FDP (μg/ml)	0–5	2.37 ± 0.66	70.15 ± 30.47	<0.001	117.06

### Patients With Severe COVID-19 Infection Present More Severe Systemic Inflammation and Overaugmented Ca^2+^ Reducing Function

For inflammation indicators, including ESR, CRP and IL-6, COVID-19 all patients with COVID-19 infection showed a significantly elevated state (the IL-6 level in patients with mild cases was within the normal range). Compared with the levels in the mild group, ESR (31.96 ± 11.24 vs. 84.14 ± 34.08 mm/h), CRP (44.61 ± 13.99 vs. 68.17 ± 15.98 mg/L), and IL-6 (3.77 ± 1.69) vs. 8.66 ± 2.16 pg/mL) were all significantly increased in the severe group (*p* < 0.001; Table 5). These results suggest that patients with severe COVID-19 infection present more severe systemic inflammation.

**Table 5:** Comparison of inflammatory factors, calcitonin and PCT in patients with mild and severe patients with COVID-19 infection.

	**Normal range**	**Patients with mild disease (*n* = 30)**	**Patients with severe disease (*n* = 30)**	***p*-value**	***F***
ESR (mm/h)	0–20	31.96 ± 11.24	84.14 ± 34.08	<0.001	70.769
CRP (mg/L)	0–10	44.61 ± 13.99	68.17 ± 15.98	<0.001	1.433
IL-6 (pg/mL)	0–7	3.77 ± 1.69	8.66 ± 2.16	<0.001	6.231
PCT (ng/mL)	0–0.1	0.04 ± 0.02	1.17 ± 0.56	<0.001	208.606
Calcitonin (ng/mL)	0–0.028	0.04 ± 0.02	1.07 ± 0.58	<0.001	160.418

Because of the presence of coagulation dysfunction and the significant decrease in Ca^2+^ concentration in patients with severe disease, we then examined the levels of PCT and calcitonin in the patients. The results showed that the PCT and calcitonin levels of all patients with COVID-19 infection were significantly higher than the normal range, which may explain why the serum Ca^2+^ levels of COVID-19 patients were significantly reduced. Compared with the mild group, the severe group had higher levels of PCT (0.04 ± 0.02 vs. 1.17 ± 0.56 ng/mL) and calcitonin (0.04 ± 0.02 vs. 1.07 ± 0.58 ng/mL; *p* < 0.001; Table 5), which may explain why patients with severe disease have lower Ca^2+^ and more severe coagulation dysfunction than those with mild disease.

### Decreased Ca^2+^, Coagulation Dysfunction, and Inflammation Indicators in Patients With COVID-19 Infection Are Significantly Correlated

**Figure 2:**
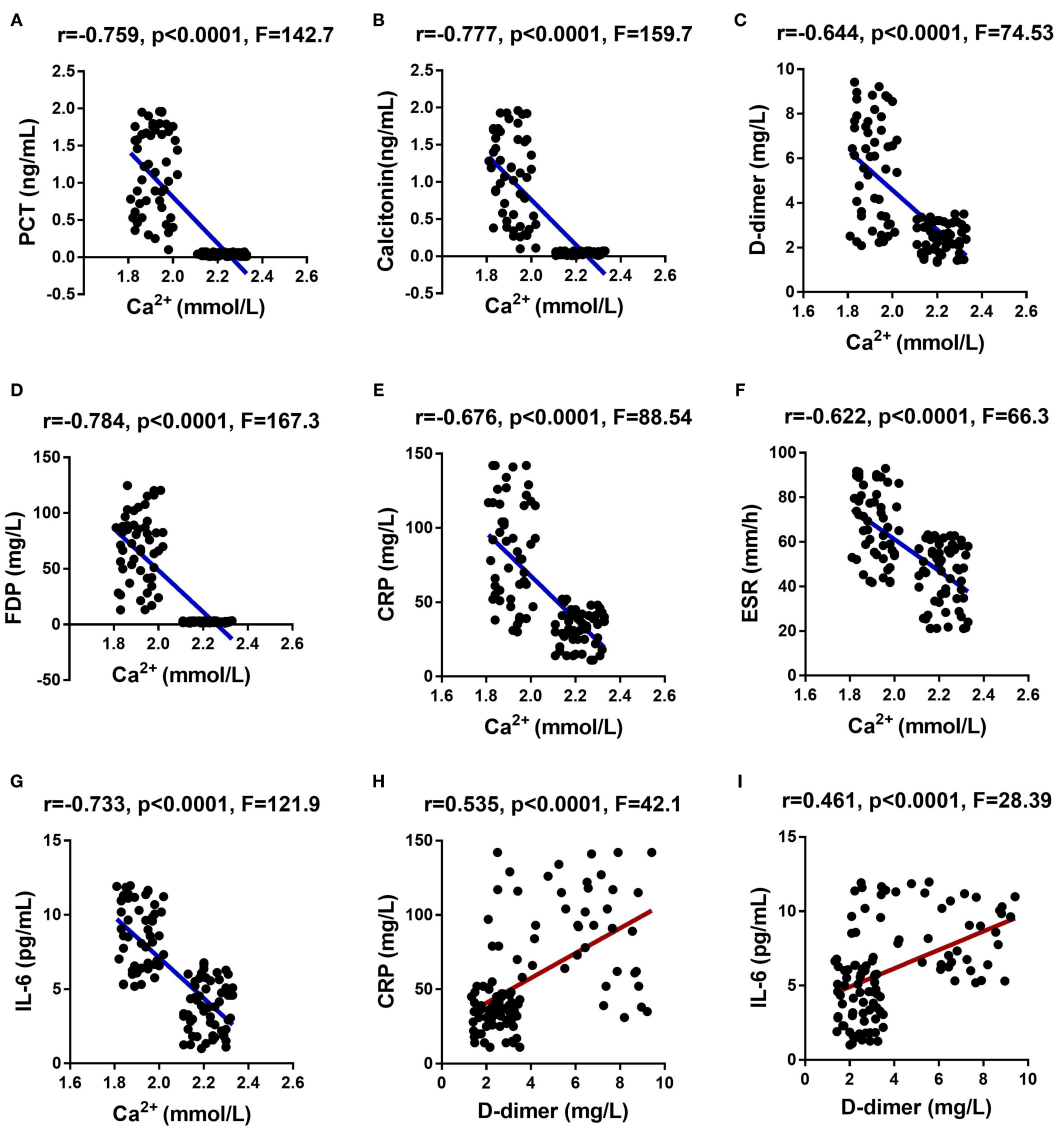
Correlation analysis among the indicators related to the reduction of Ca^2+^, coagulation function-related indicators, and inflammation indicators in COVID-19 patients. Ca^2+^ showed a significant negative correlation with PCT **(A)**, calcitonin **(B)**, D-dimer **(C)**, FDP **(D)**, ESR **(E)**, CRP **(F)**, and IL-6 **(G)**. D-dimer showed a significant positive correlation with CRP **(H)** and IL-6 **(I)**.

Finally, we analyzed the correlation between the indicators related to the reduction of Ca^2+^ (including serum Ca^2+^, PCT and calcitonin), coagulation function-related indicators (including D-dimer and PFDP), and inflammation indicators (ESR, CRP, and IL-6) in COVID-19 patients. The analysis results showed that the serum Ca^2+^ of COVID-19 patients was significantly negatively correlated with PCT, calcitonin, D-dimer, PFDP, ESR, CRP, and IL-6 (Figures 2A–G and Supplementary Table 1). The coagulation function-related indicator D-dimer had a significant positive correlation with CRP and IL-6 in COVID-19 patients (Figures 2H,I and Supplementary Table 1).

On the other hand, PT (s), PT (%), APTT (s), TT (s), INR, Fibrinogen (g/L), AT-3 (%), D-dimer (mg/L), and FDP (μg/ml) were regarded as a same variable data set and canonical correlation analysis was performed between this variable data set with other data sets, including a data set of Ca^2+^ and calcitonin and another data set of IL6, PCT, ESR, and CRP. The analysis results provided direct evidence that coagulation dysfunction was significantly related to decreased blood calcium and increased inflammation (Supplementary Tables 2, 3).

These results revealed systemic pathological changes in COVID-19 patients, and they were all correlated, including decreased Ca^2+^, coagulation dysfunction, and systemic inflammation. Disorders of metabolic function, abnormal biochemical tests, and changes in blood white blood cells present in COVID-19 patients may be caused by the decreased blood calcium and coagulation dysfunction.

## Discussion

In the present study, we first analyzed the difference between the clinical characteristics and blood cell classification of patients with mild and severe COVID-19 infection. We ruled out death cases and all the patients in the present study recovered from the infection. The data from the test results of COVID-19-infected patients were used to determine when severe symptoms appeared in the severe group. The mild group did not have severe symptoms throughout the entire course of infection.

Our data show that patients with severe disease tend to be older than patients with mild COVID-19 infection. Patients with severe disease have abnormally increased levels of white blood cells, which is consistent with previous studies (18, 19). A previous report showed that the percentage and count of monocytes in patients with mild infection are higher than those in healthy adults (20). However, our study shows that the level of monocytes in the blood is not significantly different between patients with mild and severe COVID-19 infection, and both are within the normal range. Our results also show that patients with severe disease exhibit a remarkable increase in neutrophils and a decrease in lymphocytes, while those with mild disease present neutrophils and lymphocytes in the normal range. These changes indicate that a very pronounced inflammatory response has occurred in the body of patients with severe COVID-19 infection and that the immune system suffers damage (21).

COVID-19 infection causes systemic responses in the body, including the liver (22, 23), kidney (22, 24, 25), heart (24, 26), and even the brain (27, 28). Consistent with previous reports (29, 30), we found that patients with severe COVID-19 infection have significantly increased ALT, AST, ALP, TBIL, and DBIL levels, especially AST levels, which are much higher than the normal range. The level of BUN is also significantly increased in patients with severe COVID-19 infection. We need to pay more attention to heart damage in patients with COVID-19 infection because the levels of myocardial enzymes, including CK, LDH, CK-MB, proBNP, and Myo (especially LDH and proBNP), are extremely high in patients with severe COVID-19 infection. These changes are signs of cardiac dysfunction and even heart failure (31, 32). Overall, these data reflect how patients with severe COVID-19 infection have a phenotype of systemic metabolic dysfunction, and may have damage to the liver, kidney, and heart.

Some metabolic diseases, including hypertension and diabetes mellitus, significantly affect the prognosis of COVID-19 infected patients. Hypertensive patients may have a higher risk of COVID-19 infection (33). Evidence showed that different blood type of COVID-19 hypertensive patients has different inflammatory and thrombosis status (34), which seems to be attributed to the ABO blood type may affect the coagulation process (35). The homeostasis of glucose influences the prognosis of COVID-19 infected patients with diabetes (36, 37), and hyperglycemia leads to severe inflammatory response in COVID-19 infected patients (38). The incidence of severe COVID-19 infection was significantly higher in diabetic patients compared with non-diabetic patients (39). In the present study, the data of patients with mild or severe COVID-19 infection accompanied by diabetes or hypertension were also collected. However, due to the small number of cases in the current study, the influence of hypertension or diabetes on abnormal coagulation function in COVID-19 infected patients was not further explored, but this aspect is worthy of close attention.

Ca^2+^ plays an important role in maintaining heart function and coagulation function (17, 40, 41). When testing serum electrolytes, we found that the levels of Na^+^, K^+^, and Cl^−^ were all normal in COVID-19 infected patients, although the level of Ca^2+^ in patients with severe COVID-19 infection was significantly reduced. Coagulation dysfunction may continue to alternate during COVID-19 infection. The comparison of coagulation function-related indicators of patients with mild and severe COVID-19 infection revealed that patients with severe disease had prolonged PT, lowered fibrinogen, and decreased AT-3 activity as well as extremely high D-dimer and FDP.

The above changes in coagulation function-related indicators indicate that as the severity of the disease increases, the microthrombotic load caused by the activation of the coagulation system gradually increases, while the activation and consumption of the anticoagulation system are more serious. Primary and secondary hyperfibrinolysis and bleeding tendency occur. The above characteristics indicate that the coagulation system of patients with severe COVID-19 infection presents a hypercoagulable state and microthrombosis, accompanied by activation of the anticoagulation system and consumption of anticoagulants. These features are clearly in line with the pathophysiological process of DIC (42, 43). As reported, COVID-19 patients often experience embolization and bleeding of vital organs and die of multiple organ dysfunction (3).

Coagulation dysfunction may be the reason why patients with severe COVID-19 infection have phenotypes that present systemic metabolic dysfunction and damage to other vital organs in addition to the lungs. We observed that the levels of inflammation indicators in severe COVID-19-infected patients, including ESR, CRP, and IL-6, were significantly increased. These changes in inflammation indicators have also been reported by other studies (44). Coagulation dysfunction may be the reason why damage to the liver, kidney, and heart occurs.

This and other reports (13, 45) confirmed that the Ca^2+^ concentration was reduced in patients with severe COVID-19 infection, so we detected the levels of PCT and calcitonin in the patients. In patients with severe COVID-19 infection, the decreased Ca^2+^ level corresponded to significantly increased levels of PCT and calcitonin in the blood.

At the end of this study, we conducted correlation analysis of the indicators related to the reduction of Ca^2+^, coagulation function, and inflammation in COVID-19 patients. These analyses include correlation analysis between Ca^2+^ and other indicators, and correlation analysis between D-dimer and CRP and IL-6. As we speculated, Ca^2+^ was significantly negatively correlated with PCT, calcitonin, D-dimer, PFDP, ESR, CRP and IL-6, while D-dimer was positively correlated with CRP and IL-6. We speculate that the abnormality of blood coagulation function may be caused by metabolic function disorders and abnormal biochemical tests in patients with severe COVID-19 infection. Coagulation dysfunction may also be an important factor leading to the death of COVID-19 patients because DIC often leads to embolism and bleeding in vital organs, and multiple organ dysfunction (46, 47).

Our study is not exempt from limitations. Out sample size was not large enough. Many studies have suggested the disorder of coagulation function and decreased calcium ions in COVID-19 patients; we further analyzed and discussed this theme but did not carry out the basic research for further exploration. Thereby, this aspect requires further study in order to be confirmed. Again, association between hypertension or diabetes and coagulation function in COVID-19 patients is worth exploring. On the other hand, all the patients we selected were eventually cured and discharged from the hospital, so it is worthwhile to perform further studies in COVID-19 patients without excluding the patients who eventually died.

## Conclusions

Our data confirmed that patients with severe COVID-19 infection present significant metabolic and coagulation dysfunctions, a sharp increase in serum inflammatory factors, calcitonin and PCT levels, and a significant decrease in Ca^2+^ concentration. Decreased Ca^2+^ and coagulation dysfunction in COVID-19 patients have significant correlations with inflammation. The evidence from our study may provide a better understanding of COVID-19 infection. In the treatment and monitoring of COVID-19 infected patients, attention should be devoted to the changes in the coagulation function and Ca^2+^ levels.

## Data Availability Statement

The original contributions presented in the study are included in the article/Supplementary Material, further inquiries can be directed to the corresponding author/s.

## Ethics Statement

The studies involving human participants were reviewed and approved by the ethics committee of Wuhan Jin Yin-tan Hospital. Written informed consent for participation was not required for this study in accordance with the national legislation and the institutional requirements. Written informed consent was not obtained from the individual(s) for the publication of any potentially identifiable images or data included in this article.

## Author Contributions

XQ, MW, HK, and WD drafted the manuscript. XQ and MW collected all the data. CW conducted the correlation analysis of the data. NJ, MH, TL, XW, and JW respectively, analyzed the data of the five tables in this study. WW and CH applied for the ethics of Wuhan Jin Yin-tan Hospital. YLi provided a lot of advice on this study, guided the manuscript writing, and revised the manuscript. YLiu and JT conducted the laboratory detection and applied for data disclosure from General Project of Jiangsu Provincial Health Commission. All authors contributed to the article and approved the submitted version.

## Conflict of Interest

The authors declare that the research was conducted in the absence of any commercial or financial relationships that could be construed as a potential conflict of interest.
