# Supplementary material for: Abnormal Coagulation Function of Patients With COVID-19 Is Significantly Related to Hypocalcemia and Severe Inflammation
Source: Front Med (Lausanne). 2021 Jun 16;8:638194. doi: 10.3389/fmed.2021.638194 (PMC8242574; doi:10.3389/fmed.2021.638194)
Supplement: Supplementary file 1 [file Data_Sheet_1.docx]

**Abnormal coagulation function of patients with** **COVID-19 is significantly related to hypocalcemia and severe inflammation**

Xu Qi ^1, #^, Mingjie Wu^3, #^, Hui Kong ^1,^ Wenqiu Ding ^1^, Chaojie Wu ^1^, Ningfei Ji ^1^, Mao Huang ^1^, Tiantian Li ^1^, Xinyu Wang ^1^, Jingli Wen ^1^, Wenjuan Wu ^2^, Chaolin Huang ^2^, Yu Li ^4*^, Yun Liu ^1*^, Jinhai Tang ^1*^

^1^ Department of Respiratory Medicine, The First Affiliated Hospital of Nanjing Medical University, Nanjing 210029, China

^2^ Division of Intensive Care Unit, Wuhan Jin Yin-tan Hospital, Wuhan, China

^3^ The Third Clinical Medical College, Nanjing University of Chinese Medicine, Nanjing 210023, China

^4^ School of Medicine and Holistic Integrative Medicine, Nanjing University of Chinese Medicine, Nanjing 210023, China

^#^ Equally contributed to this work.

^*^Correspondence to: Yu Li (liyu@njucm.edu.cn), Yun Liu (E-mail: E-mail: 79321531@qq.com), and Jinhai Tang (740830142@qq.com).

**Supplementary Tables**

**Supplementary Table 1.** The Pearson correlation analysis between indicated factors in mild and severe COVID-19 infected patients

| **Pearson**  **Correlation** | | Ca (mmol/L) | PT (s) | APTT (s) | TT（s） | INR | Fib (g/L) | AT-3(%) | D-dimer (mg/L) | calcitonin(ng/mL) | IL-6 (pg/mL) | Neutrophil (10^9/L) |
| --- | --- | --- | --- | --- | --- | --- | --- | --- | --- | --- | --- | --- |
| Ca (mmol/L) | Pearson Correlation | 1 | -.466^**^ | -.181 | .080 | -.054 | .445^**^ | .779^**^ | -.645^**^ | -.778^**^ | -.735^**^ | -.326^**^ |
|  | Sig. (2-tailed) |  | .000 | .063 | .411 | .581 | .000 | .000 | .000 | .000 | .000 | .001 |
| PT (s) | Pearson Correlation | -.466^**^ | 1 | -.015 | .078 | -.138 | -.327^**^ | -.446^**^ | .210^*^ | .388^**^ | .383^**^ | .014 |
|  | Sig. (2-tailed) | .000 |  | .876 | .424 | .156 | .001 | .000 | .030 | .000 | .000 | .889 |
| APTT (s) | Pearson Correlation | -.181 | -.015 | 1 | .074 | .174 | -.048 | -.193^*^ | .074 | .214^*^ | .195^*^ | .099 |
|  | Sig. (2-tailed) | .063 | .876 |  | .450 | .073 | .624 | .047 | .451 | .027 | .044 | .309 |
| TT（s） | Pearson Correlation | .080 | .078 | .074 | 1 | .002 | -.019 | .002 | -.047 | -.127 | -.093 | -.040 |
|  | Sig. (2-tailed) | .411 | .424 | .450 |  | .985 | .844 | .987 | .631 | .194 | .339 | .686 |
| INR | Pearson Correlation | -.054 | -.138 | .174 | .002 | 1 | -.080 | -.136 | .029 | .102 | .048 | -.101 |
|  | Sig. (2-tailed) | .581 | .156 | .073 | .985 |  | .412 | .164 | .767 | .296 | .621 | .302 |
| Fib (g/L) | Pearson Correlation | .445^**^ | -.327^**^ | -.048 | -.019 | -.080 | 1 | .396^**^ | -.241^*^ | -.338^**^ | -.362^**^ | -.113 |
|  | Sig. (2-tailed) | .000 | .001 | .624 | .844 | .412 |  | .000 | .012 | .000 | .000 | .248 |
| AT-3(%) | Pearson Correlation | .779^**^ | -.446^**^ | -.193^*^ | .002 | -.136 | .396^**^ | 1 | -.495^**^ | -.646^**^ | -.715^**^ | -.318^**^ |
|  | Sig. (2-tailed) | .000 | .000 | .047 | .987 | .164 | .000 |  | .000 | .000 | .000 | .001 |
| D-dimer (mg/L) | Pearson Correlation | -.645^**^ | .210^*^ | .074 | -.047 | .029 | -.241^*^ | -.495^**^ | 1 | .507^**^ | .461^**^ | .313^**^ |
|  | Sig. (2-tailed) | .000 | .030 | .451 | .631 | .767 | .012 | .000 |  | .000 | .000 | .001 |
| calcitonin(ng/mL) | Pearson Correlation | -.778^**^ | .388^**^ | .214^*^ | -.127 | .102 | -.338^**^ | -.646^**^ | .507^**^ | 1 | .657^**^ | .261^**^ |
|  | Sig. (2-tailed) | .000 | .000 | .027 | .194 | .296 | .000 | .000 | .000 |  | .000 | .007 |
| IL-6 (pg/mL) | Pearson Correlation | -.735^**^ | .383^**^ | .195^*^ | -.093 | .048 | -.362^**^ | -.715^**^ | .461^**^ | .657^**^ | 1 | .235^*^ |
|  | Sig. (2-tailed) | .000 | .000 | .044 | .339 | .621 | .000 | .000 | .000 | .000 |  | .015 |
| Neutrophil (10^9/L) | Pearson Correlation | -.326^**^ | .014 | .099 | -.040 | -.101 | -.113 | -.318^**^ | .313^**^ | .261^**^ | .235^*^ | 1 |
|  | Sig. (2-tailed) | .001 | .889 | .309 | .686 | .302 | .248 | .001 | .001 | .007 | .015 |  |
| **. Correlation is significant at the 0.01 level (2-tailed). *. Correlation is significant at the 0.05 level (2-tailed). | | | | | | | | | | | | |

**Supplementary Table 2.** The Canonical correlation analysis between coagulation dysfunction and decreased blood calcium in mild and severe COVID-19 infected patients

| **Canonical Correlations Settings** | | | | | | | |
| --- | --- | --- | --- | --- | --- | --- | --- |
| Set 1 Variables | | | PT (s), PT (%), APTT (s), TT (s), INR, Fibrinogen (g/L), AT-3 (%), D-dimer (mg/L), and FDP (ug/ml) | | | | |
| Set 2 Variables | | | Ca2+ and calcitonin | | | | |
| **Canonical Correlations** | | | | | | | |
|  | Correlation | Eigenvalue | Wilks Statistic | F | Num D.F | Denom D.F. | Sig. |
| 1 | .903 | 4.418 | .175 | 14.811 | 18.000 | 192.000 | .000 |
| 2 | .224 | .053 | .950 | .642 | 8.000 | 97.000 | .741 |
| H0 for Wilks test is that the correlations in the current and following rows are zero | | | | | | | |

**Supplementary Table 3.** The Canonical correlation analysis between coagulation dysfunction and increased inflammation in mild and severe COVID-19 infected patients

| **Canonical Correlations Settings** | | | | | | | |
| --- | --- | --- | --- | --- | --- | --- | --- |
| Set 1 Variables | | | PT (s), PT (%), APTT (s), TT (s), INR, Fibrinogen (g/L), AT-3 (%), D-dimer (mg/L), and FDP (ug/ml) | | | | |
| Set 2 Variables | | | IL6, PCT, ESR, and CRP | | | | |
| **Canonical Correlations** | | | | | | | |
|  | Correlation | Eigenvalue | Wilks Statistic | F | Num D.F | Denom D.F. | Sig. |
| 1 | .885 | 3.596 | .169 | 5.968 | 36.000 | 353.999 | .000 |
| 2 | .392 | .181 | .777 | 1.045 | 24.000 | 276.130 | .408 |
| 3 | .243 | .063 | .918 | .601 | 14.000 | 192.000 | .862 |
| 4 | .157 | .025 | .975 | .407 | 6.000 | 97.000 | .873 |
| H0 for Wilks test is that the correlations in the current and following rows are zero | | | | | | | |
