# Supplementary figures and images for: Abnormal Coagulation Function of Patients With COVID-19 Is Significantly Related to Hypocalcemia and Severe Inflammation
Source: Front Med (Lausanne). 2021 Jun 16;8:638194. doi: 10.3389/fmed.2021.638194 (PMC8242574; doi:10.3389/fmed.2021.638194)

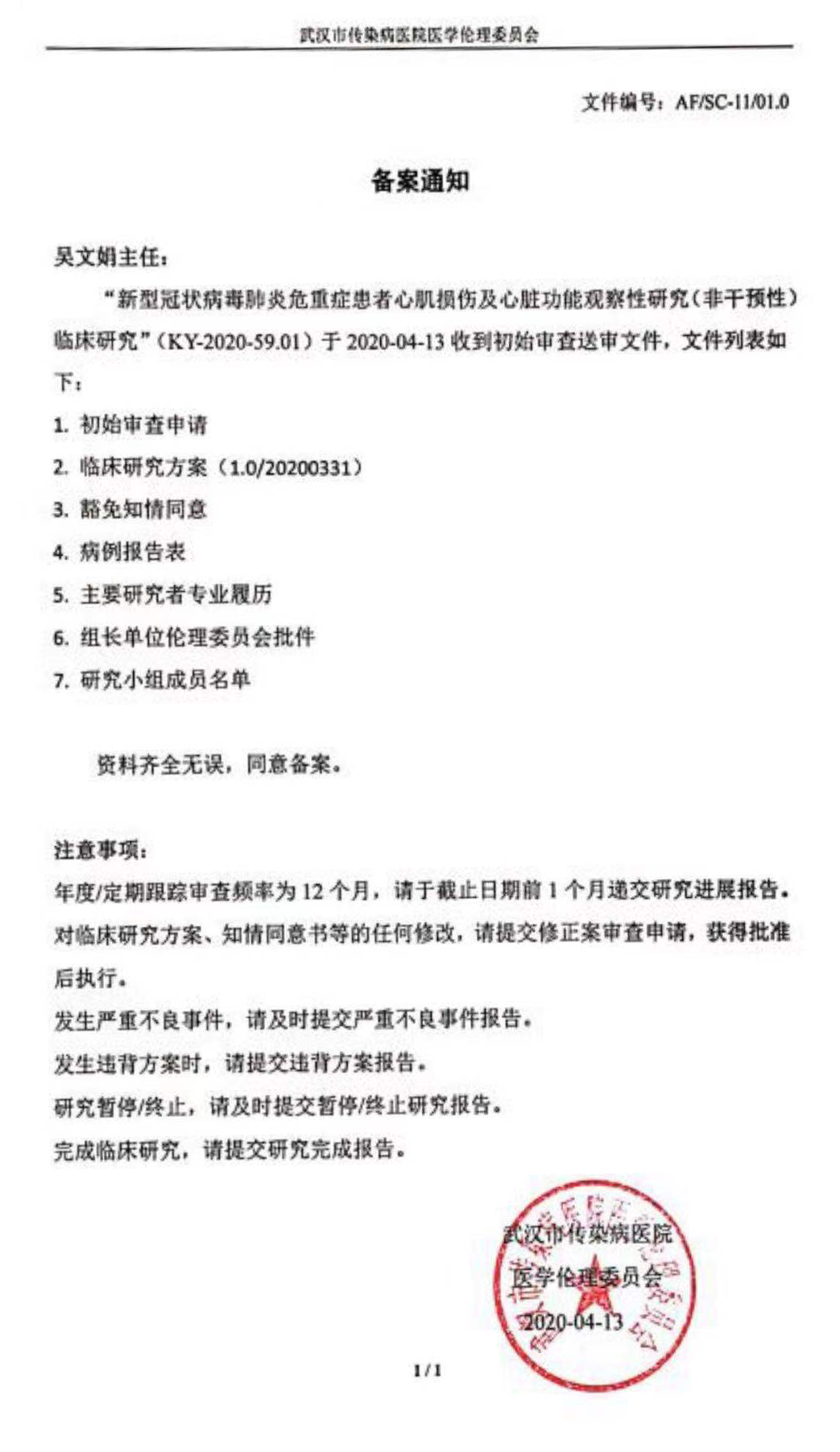

Supplement: Supplementary file 3 [file Image_1.JPEG]
